# Validation of systems biology derived molecular markers of renal donor organ status associated with long term allograft function

**DOI:** 10.1038/s41598-018-25163-8

**Published:** 2018-05-03

**Authors:** Paul Perco, Andreas Heinzel, Johannes Leierer, Stefan Schneeberger, Claudia Bösmüller, Rupert Oberhuber, Silvia Wagner, Franziska Engler, Gert Mayer

**Affiliations:** 1 Department of Internal Medicine IV (Nephrology and Hypertension), Medical University Innsbruck, Innsbruck, Austria; 2 emergentec biodevelopment GmbH, Vienna, Austria; 3 Department of Internal Medicine III (Division of Nephrology and Dialysis), Medical University of Vienna, Vienna, Austria; 4 Center of Operative Medicine, Department of Visceral Transplant and Thoracic Surgery, Medical University Innsbruck, Innsbruck, Austria; 5 Department of General, Visceral and Transplant Surgery, University of Tübingen, Tübingen, Germany

## Abstract

Donor organ quality affects long term outcome after renal transplantation. A variety of prognostic molecular markers is available, yet their validity often remains undetermined. A network-based molecular model reflecting donor kidney status based on transcriptomics data and molecular features reported in scientific literature to be associated with chronic allograft nephropathy was created. Significantly enriched biological processes were identified and representative markers were selected. An independent kidney pre-implantation transcriptomics dataset of 76 organs was used to predict estimated glomerular filtration rate (eGFR) values twelve months after transplantation using available clinical data and marker expression values. The best-performing regression model solely based on the clinical parameters donor age, donor gender, and recipient gender explained 17% of variance in post-transplant eGFR values. The five molecular markers EGF, CD2BP2, RALBP1, SF3B1, and DDX19B representing key molecular processes of the constructed renal donor organ status molecular model in addition to the clinical parameters significantly improved model performance (p-value = 0.0007) explaining around 33% of the variability of eGFR values twelve months after transplantation. Collectively, molecular markers reflecting donor organ status significantly add to prediction of post-transplant renal function when added to the clinical parameters donor age and gender.

## Introduction

Short-term renal allograft survival increased continuously during the last decades but the rate, at which transplants are lost long term remained disappointingly stable at a high level^1^. As the pathophysiology of this process is probably complex and hitherto only incompletely understood, prognostic markers available lack sensitivity and/or specificity, and treatments applied are often not successful^2^. Next to postoperative and procedure related complications like cold ischemia time, infections, rejection episodes, or the toxicity of immunosuppressive therapy, the “quality” of the donor organ has often also been associated with mid- to long term transplant survival. A proof of concept being the fact that living donation provides superior results when compared to deceased donor transplantation^1^. Unfortunately especially for deceased donors the exact procedure how to optimally describe “organ quality” is still under discussion and this uncertainty has important clinical consequences. Rejection of allocated organs by transplant centres based on opinion, rather than evidence leads to an unjustified discard of a scarce resource^3^. Kidney transplant recipients, who suffer from allograft failure after transplantation, on the other hand show even greater morbidity and mortality than patients on dialysis^4^. These caveats have fostered efforts for matching donor organ quality to recipient characteristics, but this approach needs high quality data for modelling. Donor organ quality description currently follows two concepts, one using clinical information available at the time of the offer. As an example the kidney donor profile index (KDPI)^5^, which is used for organ allocation in the US, includes among other variables donor age and the last serum creatinine. Recently van Balkom *et al*. studied the proteomic signature of the preservation fluid to derive biomarkers to predict immediate postoperative transplant function^6^. Another intuitively superior way is to use information obtained from pre-implantation biopsies. Next to unresolved technical issues (wedge vs. needle biopsy, frozen section vs. paraffin embedded tissue) it has recently been shown that conventional histological workup does provide reliable information in some^7^ but not all situations^8^. Researchers have started to look into molecular signatures in the biopsy tissue that predict immediate, but also mid- to long term post-transplant renal function^9–16^. Some of these studies are hypothesis driven. Donor age for example is an often used (albeit less than perfect) surrogate for organ quality. Koppelstätter and colleagues extended this concept by showing that markers of biological age in the biopsy (telomere length and expression of cell cycle inhibitors) better than chronological donor age predict post-transplant renal function as assessed by serum creatinine one year after transplantation^17^. Günther and colleagues very recently could show that the activating cytotoxicity receptor NKG2D was associated with chronological age and was indicative for post-transplant outcome^18^. However age is only one factor in a likely complex molecular interaction phenotype that determines organ quality in a way that it affects transplant outcome. Hypothesis driven approaches looking into single markers or pathways therefore are most likely inappropriate, a problem that could be solved by studying marker combinations or panels. However, as long as the molecular pathophysiology of chronic allograft failure is not completely understood unbiased approaches like whole-genome expression data sets obtained from pre-implantation biopsies are an important data source. Strategies on the identification of biomarkers making use of computational modelling and data integration approaches seem reasonable in this context and were elegantly summarized by Wang and Sarwal^19^. Recently O’Connel and colleagues published a study on the use of transcriptomic profiling using tissue obtained from protocol biopsies taken three months after transplantation to predict long term outcome^20^.

In this study we used a data integration approach to build a molecular model reflecting pre-implantation donor organ status. This model was based on features associated with mid- to long term allograft function from published hypothesis driven research articles and from transcriptomics data sets. Bioinformatics network analysis reduced the number of genes by focusing on those, which are more likely important features in interaction networks. A major limitation of published data on biomarkers in this context is that they are usually not validated in external cohorts. We therefore finally tested our in-silico derived markers in an independent group of renal allograft recipients, in whom gene profiling data were available from pre-implantation biopsies.

## Results

**Figure 1:**
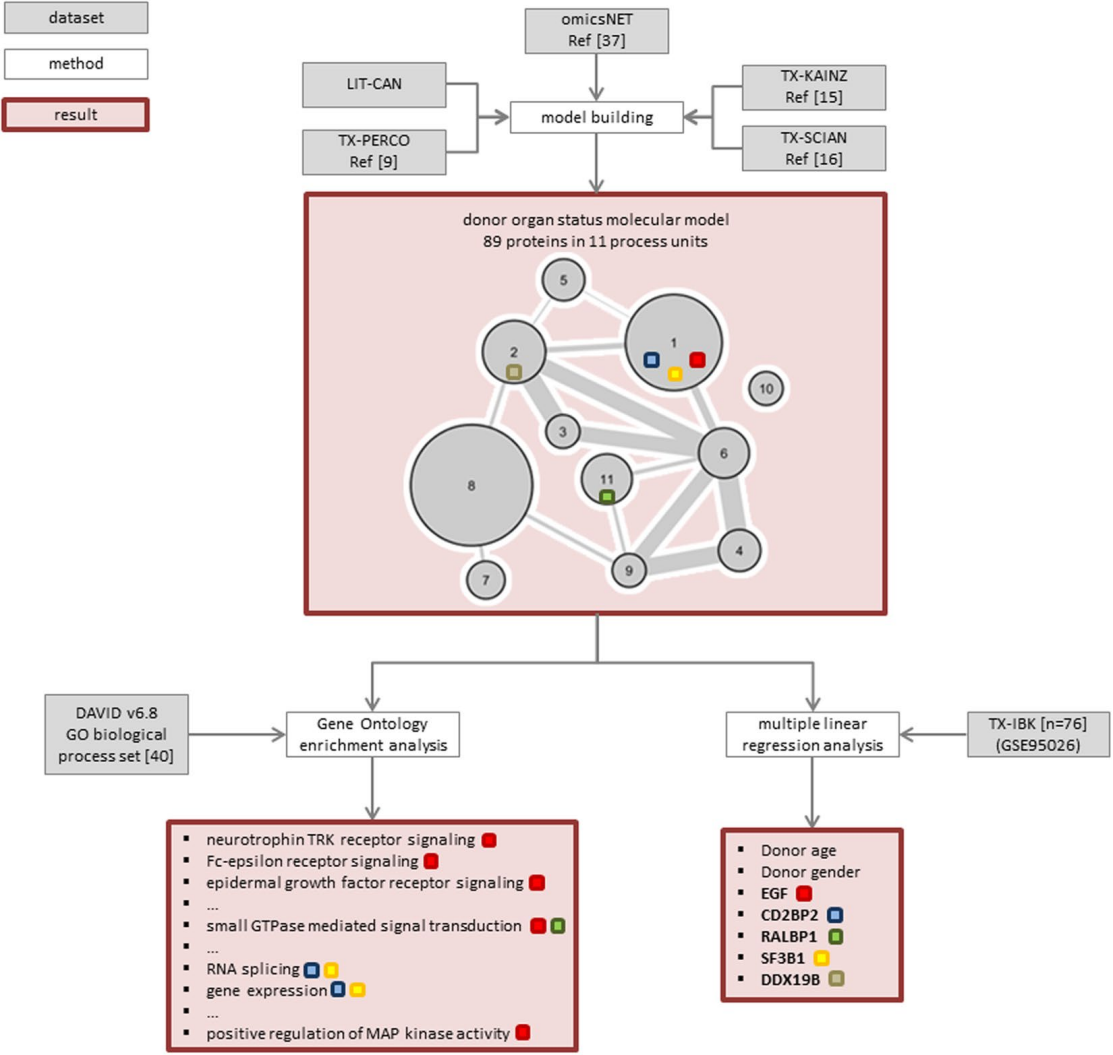
Data analysis workflow and results. Schematic representation of the data analysis workflow with used datasets, methods, and results indicated by grey, white, and red boxes respectively. Assignment of molecular markers to molecular model process units as well as enriched GO biological processes based on molecular model input features is indicated by colored squares.

Figure 1 outlines the data analysis workflow along with key results on identified molecular markers and biological processes.

### The donor organ molecular model

A set of 548 molecular features associated with renal function 12 months after transplantation could be derived based on molecular feature sets from LIT-CAN, TX-PERCO, TX-KAINZ, and TX-SCIAN. A brief description of datasets used in the analysis is listed in Table 1.

**Table 1:** Listing of datasets used.

Dataset acronym	Dataset description	Dataset use	Ref
LIT-CAN	Set of molecular features linked to chronic allograft nephropathy obtained via literature mining. Eight molecules were in the end derived from six distinct publications.	Molecular features were used as input for generating the donor organ status molecular model.	^45–50^
TX-PERCO	Transcriptomics study on renal zero-hour biopsies reporting differentially regulated genes associated with histopathological characteristics of the donor organ.	Molecular features linked to medium-term post-transplant outcome after re-analysis were used as input for generating the donor organ status molecular model.	^9^
TX-KAINZ	Transcriptomics dataset reporting on differentially expressed genes between a group with high (>=45 ml/min/1.73 m^2^) and low (<45 ml/min/1.73 m^2^) eGFR group 12 months after renal transplantation.	Molecular features were used as input for generating the donor organ status molecular model.	^15^
TX-SCIAN	Transcriptomics dataset reporting on differentially expressed genes between a group with high (>=45 ml/min/1.73 m^2^) and low (<45 ml/min/1.73 m^2^) eGFR group 12 months after renal transplantation.	Molecular features were used as input for generating the donor organ status molecular model.	^16^
omicsNET	Hybrid protein interaction network holding protein-protein interactions together with computationally inferred relations.	This biological network was used in order to generate the donor organ status molecular model.	^37^
DAVID v6.8 GO biological process set	Set of gene to GO biological process relations as stored in DAVID v6.8.	GO biological process set was used for enrichment analysis in order to identify affected biological processes based on the set of molecular features in the donor organ status molecular model.	^40^
TX-IBK	Set of 76 gene expression profiles of renal pre-implantation biopsies with clinical data of donor as well as transplant recipient at baseline and during follow up.	Expression data and clinical data were used for building multiple linear regression models in order to predict post-transplant kidney function.	GSE95026

Overview and brief description of used datasets within the present study. The specific use of the dataset is given along with the links to original publications.

LIT-CAN consisted of eight genes, the transcriptomics datasets TX-PERCO, TX-KAINZ, and TX-SCIAN held 260, 46, and 259 molecular features respectively. 391 of the molecular features shared at least one protein interaction to another member of the combined set thus forming the induced subgraph with omicsNET as the underlying biological network. Eleven clusters of highly interconnected proteins were identified by the MCODE algorithm with 89 proteins assigned to these eleven clusters. A set of 34 GO biological processes could be identified as being enriched based on the set of 89 proteins being part of the donor organ status molecular model. The top-ranked GO biological processes were the neurotrophin TRK receptor signaling pathway (FDR < 0.001), the Fc-epsilon receptor signaling pathway (FDR < 0.001), the epidermal growth factor receptor signaling pathway (FDR < 0.001), as well as the fibroblast growth factor receptor signaling pathway (FDR = 0.0027).

### Predicting post-transplant renal function

**Figure 2:**
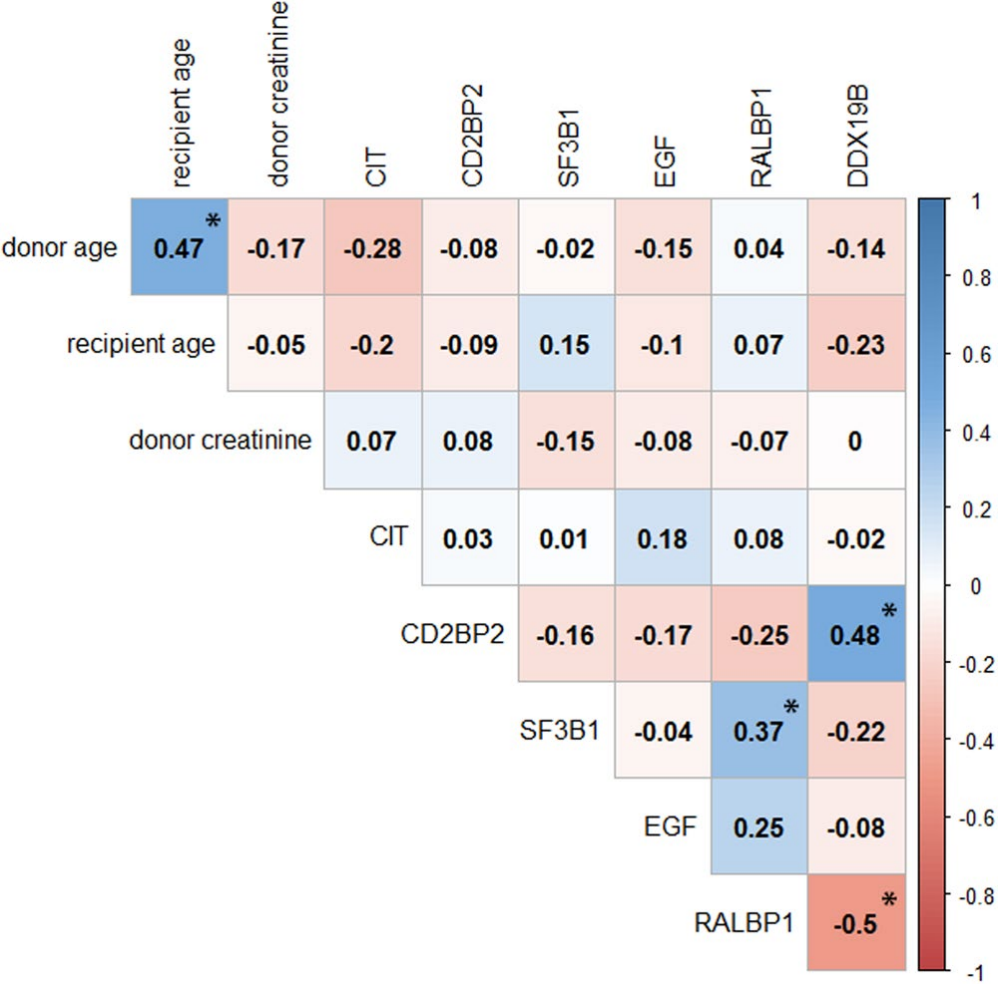
Correlation plot of continuous predictor variables. Pairwise Pearson correlations are displayed with positive and negative correlations indicated by blue and red shaded areas respectively. Significant correlations after Bonferroni correction for multiple testing are indicated by asterisks. CIT = cold ischemia time.

Average donor age in our cohort was 54 years ranging from 18 to 86. Donor age was significantly correlated to recipient age (R = 0.47, p-value < 0.0001) (see Fig. 2). The ratio of women to men in our dataset was around 1:1.5 for donors and around 1:1.7 for recipients. Average eGFR value 12 months after transplantation was 47.23 ml/min/1.73 m^2^ ranging from 6.38 ml/min/1.73 m^2^ to 100.74 ml/min/1.73 m^2^.

**Table 2:** Clinical characteristics of the TX-IBK cohort.

Parameter	TX-IBK cohort [n=76]
parameters known at the time of transplantation	
donor age [years]	54.25 (17.35)
donor gender [m/f]	46/30
last donor creatinine [mg/dl]	1.12 (0.64)
cold ischemia time [hours]	14.60 (4.88)
recipient age [years]	54.99 (12.89)
recipient gender [m/f]	48/28
transplantation number [1st/2nd]	63/13
panel reactive antibodies [0%/<20% />20%/NA]	47/8/6/15
HLA mismatches [0/1/2/3/4/5/6]	16/8/15/17/9/7/4
post-transplant parameters	
biopsy-prove rejection (yes/no/NA)	9/64/3
delayed graft function (2/1/0)	13/21/42
post-trasplant outcome parameter	
eGFR 12 months post TX [ml/min/1.73 m^2^]	47.23 (21.56)

Average values with standard deviations in brackets are given for the continuous clinical parameters donor age, last donor creatinine, cold ischemic time, recipient age, and eGFR 12 months post TX. Counts are given for the categorical variables donor gender, recipient gender, transplantation number, panel reactive antibodies, and sum of HLA mismatches as well as for the post-trasnplant parameters biopsy-proven rejection and degree of delayed graft function (2 = severe, 1 = mild, 0 = none).

The best performing linear model solely holding clinical parameters consisted of donor age, donor gender, and recipient gender and explained about 17% of variance of recipient eGFR values twelve months after transplantation (p-value = 0.0010).

The best performing linear model holding both clinical parameters and molecular markers consisted of donor age and gender along with the expression levels of the markers EGF (epidermal growth factor), CD2BP2 (CD2 cytoplasmic tail binding protein 2), RALBP1 (ralA binding protein 1), SF3B1 (splicing factor 3b subunit 1), and DDX19B (DEAD-box helicase 19B). This set of parameters explained around 33% of post-transplant eGFR values (p-value < 0.0001) thus significantly outperforming the statistical model only holding clinical parameters following analysis of variance (ANOVA) (p-value = 0.0007). Detailed model characteristics of the statistical models are given in Table 3.

**Table 3:** Multiple linear regression analysis predicting 12 months post-TX eGFR values.

Clinical model	eGFR at 12 months post TX [n = 76]		
	Parameter estimate	p-value	Adjusted R2
Intercept	55.73	<0.0001	
Donor age (per 10 years)	−3.75	0.0062	
Donor gender (m to f)	10.15	0.0352	
Recipient gender (m to f)	9.08	0.0580	
**Total**		**0.0010**	**0.17**
**Combined model**			
Intercept	−171.83	0.3734	
Donor age (per 10 years)	−2.69	0.0316	
Donor gender (m to f)	13.95	0.0028	
CD2BP2	35.41	0.0062	
SF3B1	67.29	0.0040	
EGF	16.66	0.0008	
RALBP1	−39.27	0.0007	
DDX19B	−32.18	0.0449	
**Total**		**<0.0001**	0.33 **
**Combined model plus post-transplant parameters**			
Intercept	−247.34	0.1735	
Donor age (per 10 years)	−2.27	0.0518	
Donor gender (m to f)	11.73	0.0072	
CD2BP2	34.93	0.0038	
SF3B1	65.29	0.0028	
EGF	15.17	0.0011	
RALBP1	−32.70	0.0025	
DDX19B	−23.92	0.1121	
Delayed graft function	−8.74	0.0011	
**Total**		**<0.0001**	0.42 ***

Parameter estimates, p-values and adjusted R^2^ values for the three regression models are given. Log2 marker expression values were used in the modeling analysis. The combined model was significantly better (p-value = 0.0007) than the clinical model based on ANOVA, indicated by **. Delayed graft function information significantly improved model performance (p-value < 0.0001), indicated by ***.

Significant correlations between predictor variables were identified for the molecular markers CD2BP2 and DDX19B (R = 0.48, p-value < 0.0001), SF3B1 and RALBP1 (R = 0.37, p-value = 0.0011), as well as RALBP1 and DDX19B (R = −0.5, p-value < 0.0001). As the maximum absolute Pearson correlation was not above 0.5, inclusion of selected molecular markers into one regression model was justifiable. The full correlation matrix of continuous variables is given in Fig. 2.

Whereas the add-on information on rejection episodes did not improve model performance, knowledge on the occurrence of delayed graft function significantly improved both models. The adjusted R^2^ value of the model only holding clinical parameters increased from 0.17 to 0.28 (ANOVA p-value < 0.0001) and the adjusted R^2^ value of the best performing model based on molecular markers further increased from 0.33 to 0.42 (ANOVA p-value < 0.0001). No significant association of the five markers with either delayed graft function nor rejection episodes could be found in our dataset.

## Discussion

In this study we used a systematic data integration approach to develop a molecular model of highly interconnected proteins being associated with renal function as assessed by eGFR 12 months after transplantation. Our in-silico approach reduced the number of genes of interest and in addition allowed to focus on features with a high interaction potential thus more likely reflecting core pathophysiological processes. As the genes reported in the literature and used in our modelling approach had not been validated in independent datasets we performed an additional study with this focus. Our final prediction model, holding the five selected molecular markers EGF, CD2BP2, RALBP1, SF3B1, and DDX19B on top of two clinical parameters significantly outperformed a model only based on clinical parameters. No significant correlations between molecular markers and donor age could be detected indicating that molecular markers hold complementary information to donor age. Information on delayed graft function significantly added to predictive power whereas information on rejection episodes did not further add in explaining variability of eGFR values one year after transplantation. Rejection episodes have been reported to be associated with renal outcome in the past. This is however mostly confined to rejection episodes not responsive to steroid bolus therapy, which were not observed in our cohort. The fact that rejections were rare (n = 9) and usually mild may very well be due to the fact, that no highly immunized subjects were included and subjects suffering irreversible rejection leading to graft loss within the follow up period were excluded.

Higher EGF levels were associated with higher post-transplant eGFR levels and thus with better renal function in our dataset. This is in-line with recent findings that EGF was downregulated in progressive DN^21^. The authors could further demonstrate that intrarenal EGF values are highly correlated to urinary EGF concentrations. A positive regulatory effect of EGF in progressive kidney disease has also been previously discussed by Rudnicki and colleagues^22^. This finding could be confirmed by a study from Betz and colleagues investigating EGF levels in a diabetic nephropathy animal model as well as in a cohort of normoalbuminuric type 2 diabetes patients^23^. EGF values were also found to be downregulated in donor kidney biopsies showing reduced eGFR values after transplantation in the TX-SCIAN dataset that entered our molecular model generation workflow^16^. Dosanjh and colleagues showed that members of both the hepatocyte growth factor (HGF) as well as the EGF signaling pathway are differentially expressed in renal allografts with chronic allograft nephropathy and interstitial fibrosis as well as tubular atrophy^24^.

O’Connel *et al*. collected biopsies from allograft recipients with stable renal function three months after transplantation and identified a set of 13 genes that were predictive of the development of fibrosis at one year. Although this final set did not contain EGF the latter was found to be negatively associated with the chronic allograft damage index three months after transplantation, which is in line with our findings^20^.

Surprisingly and contradictory to all other studies Rintala and colleagues demonstrated that inhibition of the EGF signalling cascade with the EGFR inhibitor erlotinib prevented chronic rejection and led to improved allograft function as their reasoning had been that EGF in part mediated chronic allograft nephropathy^25^.

As with EGF, also higher levels of CD2BP2 were associated with higher post-transplant eGFR levels indicating that reduced expression of CD2BP2 is detrimental to renal function. CD2BP2 was initially described as factor involved in T cell activation and enhancer of interleukin 2 expression^26^. CD2BP2 was however also very recently shown to be an important factor for podocyte function. Depletion of CD2BP2 in podocytes led to proteinuria and ultimately kidney function decline in a mouse model as reported by Albert and colleagues^27^.

Higher expression of RALBP1, also known as RIP1 or receptor-interacting protein 1, in our dataset as indicated by the negative parameter estimate in the statistical model were associated with lower eGFR values post-transplant and thus reduced kidney function. RALBP1 was reported as inducer of necroptosis, a novel form of cell death, which was also linked to myocardial, renal, and cerebral ischemia-reperfusion injury^28^. Enhanced expression of RALBP1 was also found to enhance cisplatin-induced nephrotoxic acute kidney injury in an *in-vitro* model by inducing necroptosis of cultured tubular cells^29^. Higher RALBP1 expression levels were also detected in an animal model of cisplatin-induced nephrotoxicity with inhibitors of necroptosis providing protection from kidney injury in this mouse model^30^.

SF3B1 encodes for a subunit of the splicing factor 3b,being involved in RNA splicing and gene expression, which was previously reported to be detectable in exosomes of transformed Madin-Darby canine kidney cells with the potential of inducing epithelial to mesenchymal transition^31^. SF3B1 was also reported as a direct target of the hypoxia inducible factor 1 alpha (HIF1A) containing a hypoxia response element in the promoter region when investigated in an animal model of cardiac hypertrophy^32^. No information on involvement in pathophysiological renal processes could be identified in scientific literature for DDX19B being involved in mRNA transport from the nucleus. This molecule might serve as sensor of deregulated transcriptional activity in deceased donor organs after brain death. Of note that DDX19B was the least significant parameter in the constructed regression model with a p-value of 0.0449. Excluding DDX19B from the model led to a drop of the adjusted R^2^ from 0.33 to 0.30 for overall model performance. Removal of either one of the other molecular markers on the contrary led to much steeper drops of adjusted R^2^ values to 0.26 down to 0.22 for overall model performance.

Most transcriptomics studies dealing with expression profiles and outcome in the renal donor setting focused on short term outcome such as delayed graft function^12–14^, while others dealt with expression patterns in the recipient after transplantation^20,33,34^. We identified three studies reporting on transcriptional changes in the donor organ being associated with long term transplant outcome^9,15,16^. Based on these transcripts complemented by genes reported in scientific literature we constructed a network-based molecular model or organ damage and validated a set of five markers in an independent dataset in the present study. Especially the mechanistic roles of EGF, CD2BP2, and RALBP1 in kidney tissue might hold the potential to also serve as targets for therapeutic intervention.

As we were primarily interested in prediction models holding parameters known at the time of transplantation, we deliberately did not include post-operative parameters in the first place. We however evaluated the impact of the two post-operative parameters rejection episodes as well as delayed graft function on performance of our generated prediction models. Delayed graft function is a known predictor of long term graft function which we also observed in our cohort^35^. Delayed graft function indeed improved model performance significantly indicating that delayed graft function holds information being complementary to the information of our selected markers. Rejection episodes interestingly did not add to prediction in our dataset with nine patients having biopsy-proven rejections in our cohort.

A limitation of our study is that no functional experiments were conducted thus further exploring the mechanistic role of the validated markers in renal tissue damage which however was not scope of this work.

In summary renal transplant donor organ expression of the five molecular markers EGF, CD2BP2, RALBP1, SF3B1, and DDX19B significantly adds in predicting post-transplant renal function when added to the clinical parameters donor age and gender.

## Methods

### Donor organ status molecular model construction

Transcriptomics studies were identified in Pubmed using the following query: *kidney transplantation [mh] AND (microarray analysis [mh] OR gene expression profiling [mh] OR High-Throughput RNA Sequencing [mh]) NOT review [publication type]*. Titles and abstracts of 242 publications were screened for gene expression studies reporting on human deceased donor kidney biopsy workup with a focus on investigating correlations of gene expression to renal function 12 months after transplantation (either based on serum creatinine or eGFR). Three studies were identified as relevant and molecular features were extracted from these publications. These three studies were the only ones using an unbiased gene expression profiling approach in donor kidney biopsies and were thus considered for further analysis in the current study. From the studies by Kainz *et al*.^15^ [TX-KAINZ] as well as Scian *et al*.^16^ [TX-SCIAN] the reported sets of transcripts were used as input for molecular model generation. The expression dataset published by Perco and colleagues [TX-PERCO] was re-analysed with respect to correlation of transcripts to twelve months post-transplant eGFR values^9^. P-values of the correlation analysis were corrected with the R package fdrtool, setting the false discovery rate to <5%^36^. All transcripts were mapped to their respective genes using the Ensembl Gene ID as common denominator. All datasets used in the integrative analysis of this work are listed in Table 1.

The set of transcripts from the omics studies was complemented by a set of molecular features reported in scientific literature described in the donor organ and having impact on medium-term graft function [LIT-CAN]. The Pubmed query *“chronic allograft nephropathy” OR “chronic allograft dysfunction” AND humans NOT review [publication type]* resulted in 1075 publications. Publications mentioning at least one gene (based on gene2pubmed, GeneRIFs, or official gene name) in title or abstract were manually reviewed in order to extract deregulated genes in the donor organ having impact on post-transplant renal function. Next, all molecular features of donor organ status affecting outcome identified above were mapped on a hybrid interaction network including protein-protein interaction data from IntAct, BioGrid, and Reactome together with computationally inferred relations^37^. The organ status-transplant outcome specific molecular interaction subgraph was extracted from the network discarding molecular features not being connected to at least one other member from the signature. This subgraph was forwarded to the Molecular Complex Detection algorithm for identifying clusters of nodes, in the following denoted as molecular processes^38,39^. These processes are characterised by an accumulation of genes with a high level of interaction and the Database for Annotation, Visualization and Integrated Discovery (DAVID) v6.8 was used to identify significantly enriched Gene Ontology (GO) biological process terms in these processes reflecting donor organ status^40^. This in silico workflow, which was developed within the European Framework 7 project SYSKID^41–43^, reduced the number of genes derived from the literature from 548 to 89. Additionally the genes remaining for validation are part of molecular processes characterized by a high level of interaction and thus most likely hold information on relevant pathophysiology.

### Independent gene expression dataset

Wedge biopsies from 78 deceased donor kidneys were obtained prior to reperfusion. Biopsy workup, RNA extraction and hybridization to Agilent Human Gene Expression 4x44 oligonucleotide microarrays was performed as described in a previous publication^44^. The study was approved by the Ethics Board of the Medical University Innsbruck. Details on the population are provided in Table 2.

The Agi4x44PreProcess R module was used for gene expression analysis. For 27 of the 78 samples, the Agilent chip version 2 was used. Raw gene expression values obtained from the two chip versions were combined into a joined dataset using shared probe identifiers. Quantile-quantile normalization was used to normalize raw mean intensity values. Summarization of probes was successively performed on the level of the human Gene Symbol. Two samples had to be excluded from further analysis based on hierarchical cluster analysis resulting in an analysis dataset of 76 samples. Gene expression data were available for 74 of the 89 molecular features in the donor organ status molecular model described above thus qualifying for inclusion in successive multiple regression analysis.

### Multiple linear regression analysis

Linear regression models were derived using all clinical parameters listed in Table 2 in order to predict post-transplant renal function given as eGFR values twelve months after transplantation. The MDRD (Modification of Diet in Renal Disease) equation was used in order to estimate GFR values. The best performing model was identified considering adjusted R^2^ values, leave-one-out cross validated (LOOCV) R^2^ values and LOOCV root-mean-square-error values.

The clinical parameters together with all molecular features of the donor organ status molecular model were used as input for delineating improved multiple regression models for predicting eGFR values twelve months after transplantation. Simulated annealing was used to identify models holding between six and eight parameters minimizing LOOCV root-mean-square error. Individual non-significant parameters were removed in a post-processing step in order to achieve statistical significance for all single parameters in the final model. The best performing regression model was compared against the regression model only holding clinical parameters using ANOVA.

Pairwise Pearson correlation coefficients were determined between molecular markers of the best performing regression model and all continuous clinical parameters listed in Table 2, namely donor and recipient age, last donor creatinine, and cold ischemia time. Bonferroni correction for multiple testing was used in order to identify significant correlations between parameters.

The primary aim of our study was to derive prediction models based solely on data available at the time of transplantation. In addition we were interested to decipher if postoperative complications like delayed graft function or acute rejection episodes have an impact on identified associations. We therefore analysed the clinical course of the study population with regard to the need for postoperative dialysis as an indication for delayed graft function (categorized as mild with 1–3 dialysis sessions or severe with >3 dialysis sessions performed) or the incidence of biopsy-proven acute rejections (regardless of Banff categorization). We first tested whether the molecular marker panel was capable of predicting these postoperative events and successively also determined the add-on value of postoperative parameters on predicting eGFR values one year after transplantation.

### Ethics

The study protocol and the use of surplus zero hour biopsy tissue after routine workup was approved by the local IRB. As no living donors were included no informed consent from the organ donors could be obtained.

### Data availability

Raw gene expression data used in this study are available at NCBI Gene Expression Omnibus with the accession number GSE95026.

## References

[1] Matas AJ (2015). OPTN/SRTR 2013 Annual Data Report: kidney. Am. J. Transplant.

[2] Stegall MD, Gaston RS, Cosio FG, Matas A (2015). Through a glass darkly: seeking clarity in preventing late kidney transplant failure. J. Am. Soc. Nephrol..

[3] Sung RS (2008). Determinants of discard of expanded criteria donor kidneys: impact of biopsy and machine perfusion. Am. J. Transplant..

[4] Rouchi AH, Mahdavi-Mazdeh M (2016). When is Transplantation with a ‘Marginal Kidney’ Justifiable?. Ann. Transplant..

[5] Rao PS (2009). A comprehensive risk quantification score for deceased donor kidneys: the kidney donor risk index. Transplantation.

[6] van Balkom BWM (2017). Proteins in Preservation Fluid as Predictors of Delayed Graft Function in Kidneys from Donors after Circulatory Death. Clin. J. Am. Soc. Nephrol..

[7] Ruggenenti, P. *et al.* Long-term outcome of renal transplantation from octogenarian donors: A multicenter controlled study. *Am. J. Transplant.* **17**, 3159–3171 (2017). 10.1111/ajt.14459 28792681

[8] Wang CJ, Wetmore JB, Crary GS, Kasiske BL (2015). The Donor Kidney Biopsy and Its Implications in Predicting Graft Outcomes: A Systematic Review. Am. J. Transplant..

[9] Perco P (2009). Histogenomics: association of gene expression patterns with histological parameters in kidney biopsies. Transplantation.

[10] Perco P, Oberbauer R (2010). Integrative analysis of -omics data and histologic scoring in renal disease and transplantation: renal histogenomics. Semin. Nephrol..

[11] Kreepala C, Famulski KS, Chang J, Halloran PF (2013). Comparing molecular assessment of implantation biopsies with histologic and demographic risk assessment. Am. J. Transplant..

[12] Hauser P (2004). Genome-wide gene-expression patterns of donor kidney biopsies distinguish primary allograft function. Lab. Investig..

[13] Mas VR (2011). Pretransplant transcriptome profiles identify among kidneys with delayed graft function those with poorer quality and outcome. Mol. Med..

[14] Mueller TF (2008). The transcriptome of the implant biopsy identifies donor kidneys at increased risk of delayed graft function. Am J Transpl..

[15] Kainz A (2007). Gene-expression profiles and age of donor kidney biopsies obtained before transplantation distinguish medium term graft function. Transplantation.

[16] Scian MJ (2012). Identification of biomarkers to assess organ quality and predict posttransplantation outcomes. Transplantation.

[17] Koppelstaetter C (2008). Markers of cellular senescence in zero hour biopsies predict outcome in renal transplantation. Aging Cell.

[18] Günther J (2017). Identification of the activating cytotoxicity receptor NKG2D as a senescence marker in zero-hour kidney biopsies is indicative for clinical outcome. Kidney Int..

[19] Wang A, Sarwal MM (2015). Computational Models for Transplant Biomarker Discovery. Front. Immunol..

[20] O’Connell PJ (2016). Biopsy transcriptome expression profiling to identify kidney transplants at risk of chronic injury: a multicentre, prospective study. Lancet.

[21] Ju W (2015). Tissue transcriptome-driven identification of epidermal growth factor as a chronic kidney disease biomarker. Sci. Transl. Med..

[22] Rudnicki M (2009). Hypoxia response and VEGF-A expression in human proximal tubular epithelial cells in stable and progressive renal disease. Lab. Investig..

[23] Betz BB (2016). Urinary peptidomics in a rodent model of diabetic nephropathy highlights epidermal growth factor as a biomarker for renal deterioration in patients with type 2 diabetes. Kidney Int..

[24] Dosanjh A (2013). Genomic meta-analysis of growth factor and integrin pathways in chronic kidney transplant injury. BMC Genomics.

[25] Rintala JM (2014). Epidermal growth factor inhibition, a novel pathway to prevent chronic allograft injury. Transplantation.

[26] Nishizawa K, Freund C, Li J, Wagner G, Reinherz EL (1998). Identification of a proline-binding motif regulating CD2-triggered T lymphocyte activation. Proc. Natl. Acad. Sci. USA.

[27] Albert GI (2015). The GYF domain protein CD2BP2 is critical for embryogenesis and podocyte function. J. Mol. Cell Biol..

[28] Rosentreter D (2015). RIP1-Dependent Programmed Necrosis is Negatively Regulated by Caspases During Hepatic Ischemia-Reperfusion. Shock.

[29] Xu Y (2015). A Role for Tubular Necroptosis in Cisplatin-Induced AKI. J. Am. Soc. Nephrol..

[30] Tristão VR (2016). Synergistic effect of apoptosis and necroptosis inhibitors in cisplatin-induced nephrotoxicity. Apoptosis.

[31] Tauro BJ (2013). Oncogenic H-ras reprograms Madin-Darby canine kidney (MDCK) cell-derived exosomal proteins following epithelial-mesenchymal transition. Mol. Cell. Proteomics.

[32] Mirtschink P (2015). HIF-driven SF3B1 induces KHK-C to enforce fructolysis and heart disease. Nature.

[33] Famulski KS (2012). Molecular phenotypes of acute kidney injury in kidney transplants. J. Am. Soc. Nephrol..

[34] Bunnag S (2009). Molecular correlates of renal function in kidney transplant biopsies. J. Am. Soc. Nephrol..

[35] Ojo AO, Wolfe RA, Held PJ, Port FK, Schmouder RL (1997). Delayed graft function: risk factors and implications for renal allograft survival. Transplantation.

[36] Strimmer K (2008). fdrtool: a versatile R package for estimating local and tail area-based false discovery rates. Bioinformatics.

[37] Fechete R (2013). Using information content for expanding human protein coding gene interaction networks. J Comput Sci Syst Biol.

[38] Bader GD, Hogue CWV (2003). An automated method for finding molecular complexes in large protein interaction networks. BMC Bioinformatics.

[39] Heinzel A, Mühlberger I, Fechete R, Mayer B, Perco P (2014). Functional molecular units for guiding biomarker panel design. Methods Mol. Biol..

[40] Sherman BT (2007). DAVID Knowledgebase: a gene-centered database integrating heterogeneous gene annotation resources to facilitate high-throughput gene functional analysis. BMC Bioinformatics.

[41] Mayer P, Mayer B, Mayer G (2012). Systems biology: building a useful model from multiple markers and profiles. Nephrol. Dial. Transplant..

[42] Heinzel A (2015). Molecular disease presentation in diabetic nephropathy. Nephrol. Dial. Transplant.

[43] Mayer G (2017). Systems Biology-Derived Biomarkers to Predict Progression of Renal Function Decline in Type 2 Diabetes. Diabetes Care.

[44] Leierer J (2016). Metallothioneins and renal ageing. Nephrol. Dial. Transplant.

[45] Ayed K (2006). Polymorphism of the renin-angiotensin-aldosterone system in patients with chronic allograft dysfunction. Transpl. Immunol..

[46] Nikolova PN (2008). Cytokine gene polymorphism in kidney transplantation‐‐impact of TGF-beta 1, TNF-alpha and IL-6 on graft outcome. Transpl. Immunol..

[47] Azarpira N (2009). Angiotensinogen, angiotensine converting enzyme and plasminogen activator inhibitor-1 gene polymorphism in chronic allograft dysfunction. Mol. Biol. Rep..

[48] Ozaki KS (2008). Improved renal function after kidney transplantation is associated with heme oxygenase-1 polymorphism. Clin. Transplant..

[49] Israni AK (2013). Inflammation in the setting of chronic allograft dysfunction post-kidney transplant: phenotype and genotype. Clin. Transplant..

[50] Menon MC (2015). Intronic locus determines SHROOM3 expression and potentiates renal allograft fibrosis. J. Clin. Invest..

